# Advanced topic modeling with large language models: analyzing social media content from dementia caregivers

**DOI:** 10.1093/geroni/igaf120

**Published:** 2025-12-26

**Authors:** Weiqing He, Bojian Hou, Amy Zheng, Yanbo Feng, Ari Klein, Karen O’Connor, Shu Yang, Tianqi Shang, George Demiris, Graciela Gonzalez-Hernandez, Li Shen

**Affiliations:** Department of Biostatistics, Epidemiology and Informatics, Perelman School of Medicine, University of Pennsylvania, Philadelphia, Pennsylvania, United States; Department of Biostatistics, Epidemiology and Informatics, Perelman School of Medicine, University of Pennsylvania, Philadelphia, Pennsylvania, United States; Department of Biostatistics, Epidemiology and Informatics, Perelman School of Medicine, University of Pennsylvania, Philadelphia, Pennsylvania, United States; Department of Biostatistics, Epidemiology and Informatics, Perelman School of Medicine, University of Pennsylvania, Philadelphia, Pennsylvania, United States; Department of Biostatistics, Epidemiology and Informatics, Perelman School of Medicine, University of Pennsylvania, Philadelphia, Pennsylvania, United States; Department of Biostatistics, Epidemiology and Informatics, Perelman School of Medicine, University of Pennsylvania, Philadelphia, Pennsylvania, United States; Department of Biostatistics, Epidemiology and Informatics, Perelman School of Medicine, University of Pennsylvania, Philadelphia, Pennsylvania, United States; Department of Biostatistics, Epidemiology and Informatics, Perelman School of Medicine, University of Pennsylvania, Philadelphia, Pennsylvania, United States; Department of Biostatistics, Epidemiology and Informatics, Perelman School of Medicine, University of Pennsylvania, Philadelphia, Pennsylvania, United States; Department of Computational Biomedicine, Cedars-Sinai Medical Center, Los Angeles, California, United States; Department of Biostatistics, Epidemiology and Informatics, Perelman School of Medicine, University of Pennsylvania, Philadelphia, Pennsylvania, United States

**Keywords:** Natural language processing, Semantic coherence, Social media analysis, Healthcare informatics, Prompt engineering

## Abstract

**Background and Objectives:**

While traditional topic modeling methods have been applied to analyze social media content from dementia caregivers, they often struggle with semantic understanding and coherent topic generation. This study explores the direct application of large language models (LLMs) for topic modeling of caregiver tweets, aiming to leverage their advanced semantic comprehension capabilities.

**Research Design and Methods:**

We analyzed 231 870 tweets from dementia caregivers after preprocessing using ChatGPT as the primary topic modeling tool. To address context length limitations, we developed a 2-stage approach: first splitting the dataset into 226 batches of 1000 tweets each for initial topic extraction, then combining these results through a second-stage prompt for final topic synthesis. We compared our approach against 11 baseline methods, including Latent Dirichlet Allocation (LDA), Gibbs Sampling Dirichlet Multinomial Mixture Model (GSDMM), their term-weighted variants, and state-of-the-art BERTopic models. Topic quality was evaluated using Sentence-BERT-based coherence scores, and topic comprehensiveness was assessed through both ChatGPT and human expert evaluation.

**Results:**

Our LLM-based approach achieved a coherence score of 0.358, significantly outperforming all baseline methods. Traditional approaches like GSDMM (0.317) and LDA (0.320), their term-weighted variants (ranging from 0.264 to 0.302), and BERTopic variants (approximately 0.30) showed lower coherence scores. The 2-stage batching strategy effectively handled the large dataset while maintaining topic quality and representativeness. Expert evaluation confirmed the topics’ relevance to caregiver experiences and their comprehensive coverage of key themes.

**Discussion and Implications:**

This study introduces a novel methodology for applying LLMs to large-scale topic modeling tasks, demonstrating superior performance over traditional and state-of-the-art approaches. The significant improvement in coherence scores suggests that LLMs can better capture the semantic relationships within topics. Our approach addresses key challenges in context length limitations and prompt engineering, while providing more coherent and interpretable insights into caregiver experiences that can inform targeted support strategies.

Translational Significance: Analyzing social media content from caregivers presents a significant challenge due to the volume and complexity of unstructured text data. Our study demonstrates that large language models can effectively extract meaningful topics from caregiver discussions on social media, outperforming traditional analysis methods. These improved insights into caregivers’ experiences, challenges, and needs can help healthcare providers and support organizations develop more targeted interventions, inform policy makers about resource allocation, and enable the creation of more effective support systems. The methodology also provides a framework for analyzing large-scale social media data to understand other healthcare-related challenges faced by various populations.

## Introduction

The rising global prevalence of dementia represents one of the most pressing healthcare challenges of our time. Current estimates suggest an alarming increase from 55 million cases in 2023 to 115 million by 2050,^1–3^ creating an unprecedented burden not only on healthcare systems but also particularly on family caregivers. In the United States alone, approximately 6.7 million individuals live with dementia, supported by over 11 million family caregivers^4^ who face substantial psychological, physical, and financial challenges. These caregivers experience significantly higher rates of depression (33.9%) compared to those caring for individuals with other conditions,^5^^,^^6^ along with increased risks of cardiovascular diseases^7^ and financial strain, providing care valued at approximately 339.5 billion dollars annually.^4^

Understanding the multifaceted challenges faced by dementia caregivers has become increasingly possible through social media platforms, particularly Twitter (now X),^8^ where caregivers openly share their experiences, struggles, and support needs. These platforms generate vast amounts of unstructured text data that, when properly analyzed, can provide crucial insights into caregivers’ daily lives and needs. However, extracting meaningful insights from this data presents significant technical challenges, particularly in identifying coherent and semantically meaningful topics from short, informal social media posts.

Traditional approaches to analyzing social media content have relied primarily on probabilistic topic modeling techniques such as Latent Dirichlet Allocation (LDA)^9^ and the Gibbs Sampling Dirichlet Multinomial Mixture Model (GSDMM).^10^ Recent work by Feng et al.^8^ attempted to enhance these traditional models through term-weighting strategies, specifically combining “Log” and “BDC” weights to improve topic coherence in the context of dementia caregiver tweets. While their approach showed promise when evaluated using traditional coherence metrics based on word co-occurrence, these metrics themselves have limitations in capturing true semantic relationships between words.

More recent advances in natural language processing have introduced transformer-based approaches like BERTopic,^11^ which leverages sophisticated embedding techniques for topic modeling. However, these methods still face challenges in maintaining semantic consistency when dealing with specialized healthcare contexts and informal social media language. Furthermore, when evaluated using more sophisticated semantic coherence metrics based on Sentence-BERT^12^ embeddings, even term-weighted traditional models show limitations in capturing true semantic relationships between words in identified topics.

The emergence of large language models (LLMs)^13^^,^^14^ presents a promising new direction for topic modeling, offering potentially superior semantic understanding and contextual awareness. Unlike traditional probabilistic models or even transformer-based approaches, LLMs have demonstrated remarkable capabilities in understanding nuanced language and domain-specific terminology through their pre-training on vast text corpora.^15^ This makes them particularly well-suited for analyzing healthcare-related social media content, where understanding context and medical terminology is crucial.

In this article, we propose a novel approach to topic modeling using LLMs, specifically ChatGPT,^16^ to analyze tweets from dementia caregivers. To address the technical challenges of applying LLMs to large-scale social media analysis, we develop a 2-stage approach that first processes tweets in manageable batches and then synthesizes the identified topics. Crucially, we introduce a new evaluation framework based on Sentence-BERT^12^ embeddings that better captures semantic relationships between words in identified topics, providing a more meaningful assessment of topic coherence than traditional co-occurrence-based metrics.

The key contributions of this paper include:

- A novel LLM-based approach to topic modeling that directly leverages the semantic understanding capabilities of large language models for analyzing social media content.
- A 2-stage processing pipeline that effectively addresses the context length limitations of LLMs while maintaining semantic coherence in topic identification.
- A new evaluation framework based on Sentence-BERT embeddings that provides a more meaningful assessment of semantic coherence in identified topics.
- Comprehensive empirical evaluation demonstrating superior performance of our LLM-based approach compared to eleven baseline methods, including both traditional and state-of-the-art topic modeling approaches.
- Practical insights into dementia caregiver experiences derived from more semantically coherent topic identification, with implications for healthcare providers, support organizations, and policy makers.

**Figure 1.**
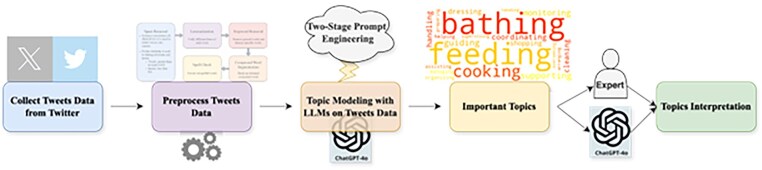
The illustration of the framework.

Our results demonstrate that LLM-based topic modeling can achieve significantly higher semantic coherence scores compared to traditional methods and their term-weighted variants, while providing more interpretable and actionable insights into caregiver experiences. These improvements in topic modeling accuracy and interpretability could lead to more effective support systems and interventions for the growing population of dementia caregivers. The illustration of our framework is shown in Figure 1.

### Related work

Recent research has explored various text analysis techniques to understand the experiences of dementia caregivers.^17-19^ Among these methods, topic modeling stands out as a powerful statistical tool for uncovering latent, or hidden, themes within large datasets. While topic modeling is widely used across various data types,^20-23^ it is particularly well-suited for analyzing text data.

Traditional probabilistic approaches like LDA^9^ and Dirichlet Multinomial Mixture^24^ (DMM) form the foundation of topic modeling. LDA assumes each document in a text corpus is composed of multiple topics, where each word is generated from a distribution over these topics. However, LDA faces challenges when applied to short texts, such as tweets, where limited word counts make it difficult to establish clear topic distributions. To address this, the DMM model was proposed as an alternative. Unlike LDA, DMM assumes that each document is associated with only one topic, making it more suitable for short and focused texts. Building on DMM, the GSDMM^10^ model was introduced, combining DMM’s single-topic assumption with Gibbs sampling^25^ to enhance topic clustering in short, noisy text.

To further enhance the performance of topic models like LDA and GSDMM on short, noisy text, techniques such as term weighting are often applied.^26^^,^^27^ Term weighting involves adjusting the importance of specific words within the text corpus to refine topic quality by minimizing the influence of high-frequency, low-information terms. One approach to term weighting assigns lower weights to common words and higher weights to rarer, more informative words, which helps focus the model on meaningful content.^8^ Additionally, weighting schemes like TF-IDF^28^ (term frequency-inverse document frequency) are used to emphasize terms that are distinctive to particular documents within a corpus. By integrating term weighting, models like LDA and GSDMM can achieve better performance and more interpretable results on short-text datasets.

Recent advances in transformer-based models have led to new topic modeling approaches. BERTopic^11^ represents a significant departure from probabilistic methods, utilizing pre-trained language models for document embedding followed by clustering. It combines BERT-based embeddings with UMAP dimensionality reduction and HDBSCAN clustering, followed by a class-based TF-IDF procedure to extract representative terms for each topic. While BERTopic has shown promise on various datasets, its performance can vary depending on the specific embedding model used and the nature of the text being analyzed.

The emergence of LLMs has opened new possibilities for topic modeling. Recent work has explored using LLMs for various aspects of topic modeling, including topic label generation^29^ and topic interpretation.^30^ Some studies have investigated using LLMs for direct topic extraction,^31^^,^^32^ though primarily in controlled settings with formal documents rather than social media content. Our work extends this emerging direction by applying ChatGPT to perform end-to-end topic modeling on caregiver tweets, developing novel techniques to handle the unique challenges of social media text while leveraging the semantic understanding capabilities of LLMs.

## Methods

### Ethical considerations

This study was conducted in strict accordance with ethical research principles and social media data usage guidelines. All data collection and analysis procedures followed Twitter’s (now X) Terms of Service and Developer Agreement regarding public data usage. The study protocol was reviewed by the Institutional Review Boards (IRBs) of both the University of Pennsylvania and Cedars-Sinai Medical Center, who determined that the research qualified for exemption under 45 CFR 46.104(d)(4)(i), as it involves the secondary analysis of publicly available data.

To protect user privacy, all tweet content was anonymized during the analysis process, with personal identifiers removed. While we analyzed public tweets, we acknowledged the sensitive nature of caregiving-related discussions and implemented additional safeguards to ensure responsible use of the data. We limited our analysis to aggregate patterns and themes, avoiding the presentation of individually identifiable information or direct quotes that could potentially compromise user privacy.

The research team completed required ethical training in human subject research and followed established guidelines for ethical social media research. Our methodology and reporting adhere to best practices for social media health research while maintaining respect for the caregiver community whose experiences inform this work.

### Data collection

Our dataset was collected through a systematic, multi-stage process designed to identify relevant tweets from potential dementia family caregivers. The collection process leveraged a deep neural network classifier that was previously developed and validated by Klein et al.^33^ To develop the classifier, 3 annotators manually labeled 10 733 tweets for whether they reported having a family member with dementia, with an inter-annotator agreement (Fleiss’ kappa) of 0.82, and the annotated tweets were used to fine-tune a BERT model specifically pre-trained on Twitter data.^34^ The classifier demonstrated robust performance with an F1 score of 0.962 (precision = 0.946, recall = 0.979) for identifying tweets that reported having a family member with dementia.^35^

Using the Twitter Streaming API, we collected 362 979 original tweets (excluding retweets) posted between May 2021 and March 2023. These tweets were collected using a comprehensive keyword strategy documented in Klein et al.,^33^ which includes 3 categories of terms: (1) dementia-related keywords (eg, “dementia,” “youngdementia,” “#yod,” “#ftd,” “alzheimer’s”, “alz,” “alzheimersdisease,” “mild cognitive impairment”), (2) keywords indicating a potential diagnosis (eg, “diagnosed,” “diagnosis,” “has,” “got,” “developed,” “with,” “from”), and (3) keywords referring to familial relationships. The complete keyword list and query construction methodology are detailed in Klein et al.^33^ This collection strategy was designed to capture discussions related to dementia family caregiving experiences while minimizing noise from unrelated conversations.

We then applied the pre-trained classifier to these 362 979 tweets, which identified 231 870 relevant tweets from 124 062 unique users. These tweets specifically indicated having a family member with dementia, thus implying potential involvement in caregiving. For example, tweets such as “I live with my father who has dementia and was considering an Apple Watch to help us find him when he wanders” or “My mom with Alzheimer’s doesn’t recognize me anymore, but still smiles when I visit” were classified as positive. Klein et al.^33^ provide additional examples and detailed classification criteria in their supplementary materials. This filtered dataset, focusing on personal experiences rather than general discussions about dementia or posts from healthcare professionals, formed the basis for our topic modeling analysis.

### Data quality verification

To ensure data quality, we implemented several verification steps:

1. We performed additional filtering beyond the initial data collection. Tweets were preprocessed (removing URLs, mentions, normalizing text) and converted to 384-dimensional embeddings using Sentence-BERT. For users posting ≥10 tweets, we calculated mean pairwise cosine similarity between their tweets. Users with similarity ≥0.6 (indicating repetitive or automated content) had only one tweet randomly selected and retained from their pool of similar tweets, removing redundant content while preserving diverse caregiver voices without selection bias.
2. Language consistency verification: All tweets were verified to be in English, as the initial Twitter API collection was restricted to English-language content following the methodology in Klein et al.^33^
3. Temporal distribution assessment: The dataset spans continuously from May 2021 to March 2023, ensuring comprehensive temporal coverage across the 23-month study period without significant gaps.
4. Geographic diversity validation: Geographic representation was achieved through Twitter’s global streaming API, which naturally captures tweets from diverse English-speaking regions worldwide without geographic restrictions.

### Data preprocessing

Despite careful initial data collection, social media content inherently contains noise, spam, and inconsistencies that could impact topic modeling quality. To address these challenges, we developed a comprehensive preprocessing pipeline that systematically improved data quality while preserving meaningful content.

Our preprocessing began with fundamental cleanup operations, removing URLs, usernames, and non-word entities while standardizing text format through lowercasing and spacing normalization. To handle duplicate and near-duplicate content, we implemented an embedding-based approach where tweets were transformed into 384-dimensional dense vectors. By computing cosine similarities between tweets from the same user, we identified and addressed redundant content. For users who posted frequently (10 or more tweets) with high content similarity (cosine similarity ≥0.6), we retained only one representative tweet, thereby preserving content diversity while reducing redundancy.

Text normalization played a crucial role in our preprocessing strategy. Using the WordNetLemmatizer from NLTK,^36^ we standardized word forms to their base representations, ensuring consistent treatment of conceptually identical terms. We then addressed the challenge of non-informative words through a 2-step approach: first applying NLTK’s standard English stopword list, then manually removing domain-specific terms including data collection keywords related to dementia and family relationships.

Informal writing styles on social media presented additional challenges, particularly in handling compound words. Using the wordsegment package,^37^ we decomposed informal compounds like “bestthing” and “newworld” into their constituent parts, improving text standardization while maintaining semantic clarity. The final step involved spelling normalization through pyspellchecker,^38^ addressing common misspellings and variant spellings that could otherwise introduce noise into our analysis.

This careful preprocessing reduced our dataset from 231 870 to 225 357 tweets, representing a modest 3% reduction in data volume while substantially improving data quality. Each processing decision was made with careful consideration of the balance between noise reduction and information preservation, ensuring that the resulting dataset would provide a strong foundation for subsequent topic modeling analysis.

### Two-stage topic modeling with ChatGPT

The analysis of large-scale social media data presents unique challenges, particularly when working with language models that have context length limitations. To overcome these constraints while leveraging the advanced semantic understanding capabilities of GPT-4 (version gpt4o-2024-08-06), we developed a 2-stage topic modeling approach that effectively processes and synthesizes large volumes of tweets while maintaining semantic coherence.

**Figure 2.**
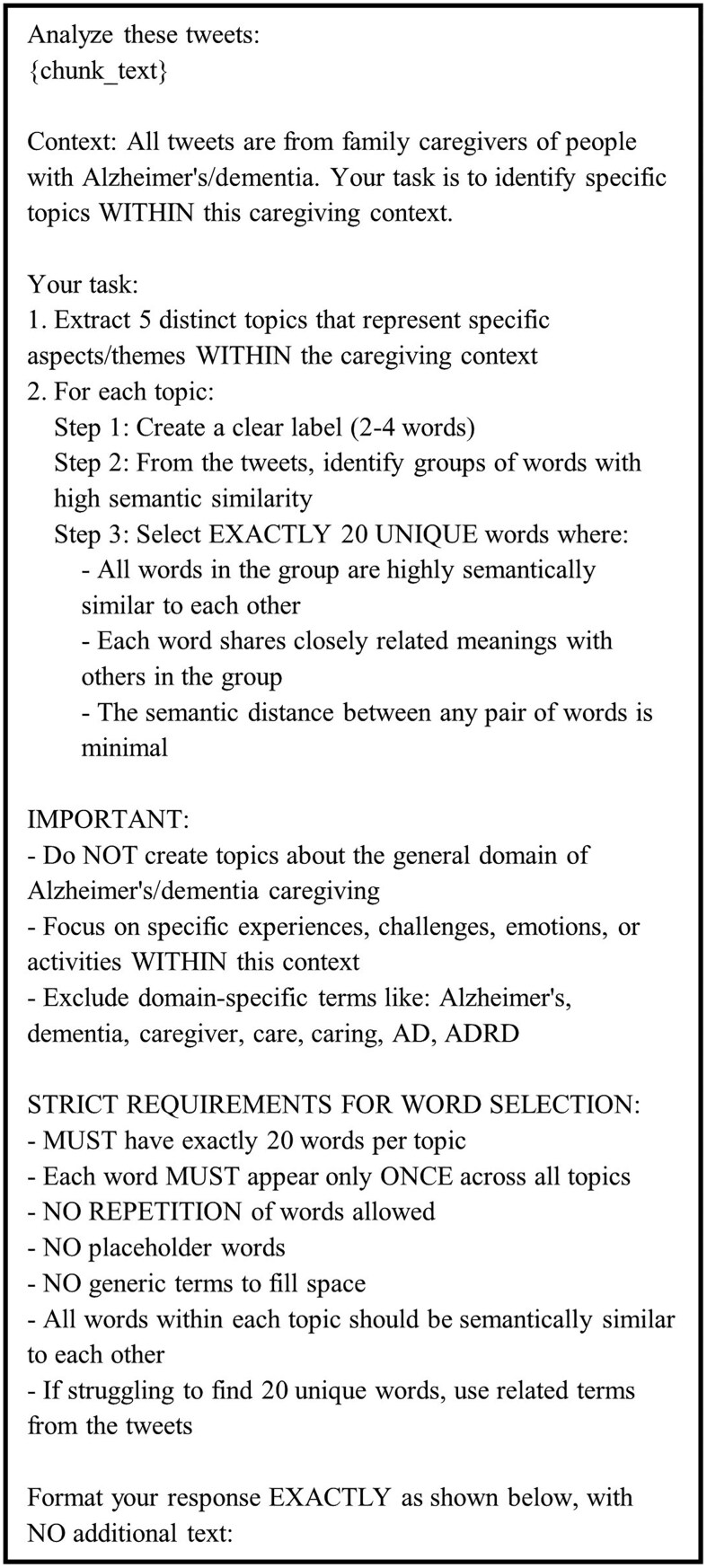
The prompt for instructing ChatGPT to extract initial topics for each batch of tweets.

We began by dividing our dataset of 225 357 tweets into manageable batches of 1000 tweets each, resulting in 226 batches. For each batch, we engaged GPT-4 in initial topic extraction using a carefully crafted prompt as shown in Figure 2. This prompt was designed to guide the model in identifying specific aspects and themes within the caregiving context while avoiding general or domain-redundant topics. We explicitly instructed the model to create 5 distinct topics, each comprising exactly 20 unique words with high semantic similarity. The prompt emphasized the importance of selecting words that share closely related meanings and minimal semantic distance, while explicitly excluding domain-specific terms like “Alzheimer’s” or “dementia” that would be redundant given the context.

**Figure 3.**
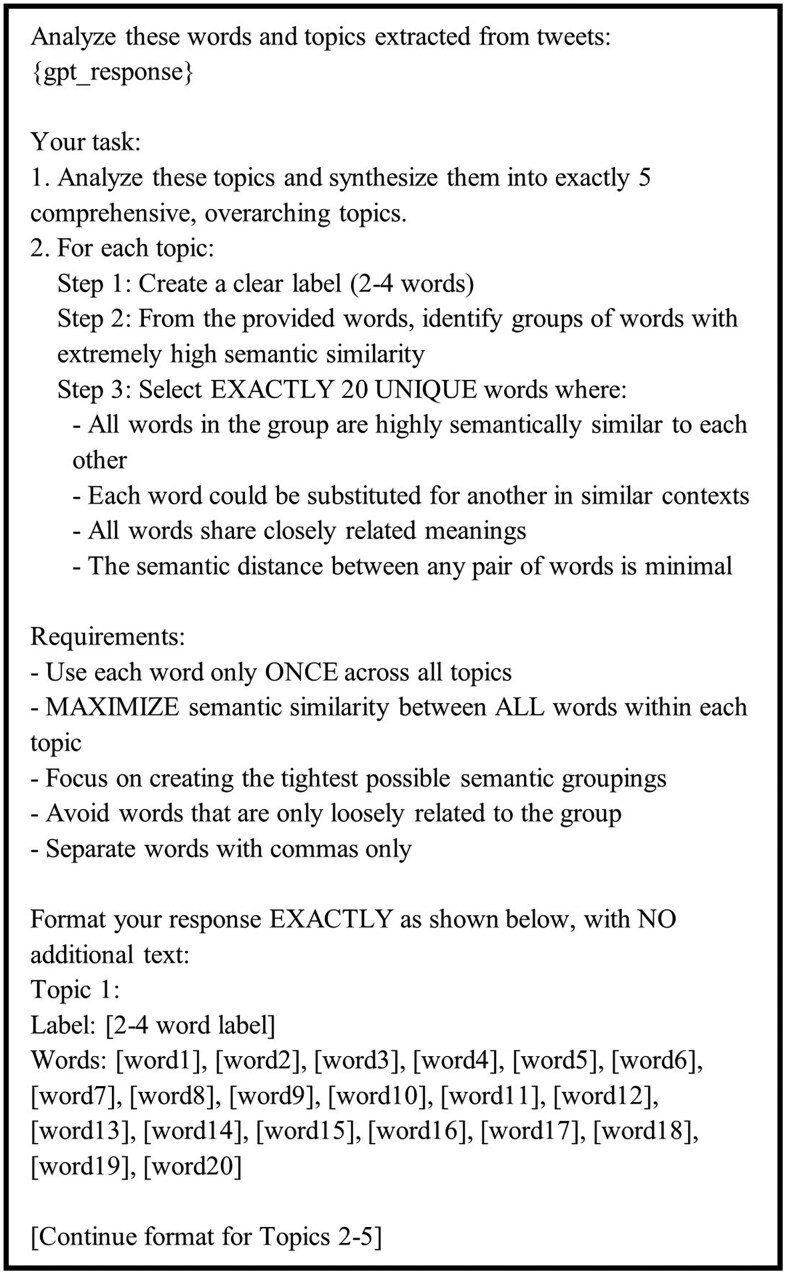
The prompt for instructing ChatGPT to synthesize all the initial extracted topics.

This initial stage of analysis generated 1130 topics across all batches (5 topics × 226 batches). To synthesize these initial topics into a coherent final set, we developed a second prompt that guided GPT-4 in combining and refining the topics as illustrated in Figure 3. This synthesis prompt emphasized the creation of comprehensive, overarching themes while maintaining strict semantic similarity requirements within each topic.

Our 2-stage approach proved effective in managing the complexity of large-scale social media analysis while maintaining semantic coherence. The first stage enabled detailed analysis of local patterns within each batch, while the second stage synthesized these patterns into global themes. Throughout both stages, we maintained strict requirements for word selection and semantic similarity, ensuring that each final topic would provide meaningful insights into specific aspects of caregiving experiences.

The iterative refinement of our prompts played a crucial role in the success of this approach. Through systematic pilot testing involving over 15 iterations, we optimized the prompts to consistently produce clear, distinct topics with minimal redundancy and high semantic coherence. To validate our choice of *K* = 5 topics, we conducted a sensitivity analysis with *K*={5, 8, 10, 13}. The coherence scores remained remarkably stable across different topic numbers (0.362, 0.349, 0.365, and 0.357, respectively), demonstrating that our approach is robust to this parameter. We selected *K* = 5 based on 3 key considerations: (1) computational efficiency, as processing 226 batches with fewer topics significantly reduces API costs and processing time; (2) interpretability, as 5 major themes provide clear, actionable insights for healthcare providers without overwhelming complexity; and (3) methodological consistency with prior work analyzing the same population,^8^ enabling direct comparison. The 20-word specification per topic follows standard practice in coherence evaluation literature,^8^ where 10-20 words optimally balance comprehensive semantic coverage with maintaining tight relationships between terms. This careful attention to prompt engineering and parameter selection helped ensure that our final output would provide valuable insights into the experiences of dementia caregivers while maintaining robust semantic relationships within each topic group.

### Evaluation metric

Evaluating the quality of extracted topics requires careful consideration of the semantic relationships between words within each topic. While traditional topic coherence metrics (eg, C_v score^8^) often rely on word co-occurrence patterns in the corpus, such approaches may not fully capture the semantic relationships in our context, particularly when dealing with topics extracted by large language models that might introduce words not present in the original corpus. To address this challenge, we developed a semantic coherence metric based on contextual word embeddings.

Our evaluation framework leverages Sentence-BERT^12^ (version all-MiniLM-L6-v2) to capture the semantic meaning of each word in its contextual space. For a given topic containing 20 words, we first transform each word $\;w_{i}\;$ into its vector representation $\;v(w_{i})\;$ using Sentence-BERT, resulting in high-dimensional (384) embeddings that capture the nuanced meanings of words. These embeddings provide a rich semantic representation that accounts for the multiple potential contexts in which each word might appear.

More concretely, for each topic  T, which consists of a bag of words $\;W=\{w_{1},\;w_{2},\;\ldots ,\;w_{20}\}$, we first embed each word using Sentence-BERT to obtain its vector representation:


$$v(w_{i})\;=\;\mathrm{SentenceBERT}(w_{i}),\;w_{i}\;\in \;W$$

where  v(wᵢ) ∈ Rᵈ  represents the  d-dimensional embedding vector of word  wᵢ.

We then compute the pairwise semantic similarity between all words in the topic using cosine similarity:


$$\mathrm{sim}(w_{i},\;w_{j})\;=\;\;\text{cos}(v(w_{i}),\;v(w_{j}))\;=\;v(w_{i})ᵀv(w_{j})/(||v(w_{i})||\;||v(w_{j})||)$$

where  ||·||  denotes the $\;L_{2}\;$ norm of a vector.

The coherence score for a topic  T  is then calculated as the average of all pairwise similarities:


$$C(T)\;=\;2/(n(n-1))\;\sum_{i<j}\;\mathrm{sim}(w_{i},w_{j})$$

where  n=|W|=20 is the number of words in the topic. The factor  2/(n(n−1)) normalizes the sum over all  n(n−1)/2 unique pairs.

The overall coherence score for a model  M  with  k  topics is computed as the average coherence across all topics:


$$C(M)=1/k\;\sum_{i=1}^{k}\;C\;(Tᵢ)$$

This metric provides a single, interpretable score that reflects the overall semantic quality of the extracted topics. Higher scores indicate that words within each topic are more semantically related, suggesting more coherent and meaningful topics. This evaluation approach offers several advantages over traditional coherence metrics. It captures deep semantic relationships through contextual embeddings, provides a fair comparison across different modeling approaches (including both traditional methods and LLM-based approaches), and yields interpretable scores bounded between 0 and 1. Moreover, by using Sentence-BERT embeddings, we can capture nuanced semantic relationships that might not be apparent from simple co-occurrence statistics, making the metric particularly suitable for evaluating topics generated through various approaches.

### Baseline methods

To evaluate the effectiveness of our LLM-based topic modeling approach, we conducted extensive comparisons against a broad range of baseline methods, from traditional probabilistic models to recent transformer-based approaches.

The fundamental challenge in analyzing social media content from dementia caregivers lies in the inherent characteristics of tweets: limited to 280 characters, these posts are semantically sparse and typically contain fewer topics compared to longer texts. While our preprocessing pipeline effectively reduced noise, the remaining general words and domain-specific terms can still obscure underlying semantic themes. This challenge motivates our comprehensive comparison against multiple baseline approaches.

Traditional probabilistic methods serve as our first category of baselines:

- LDA^9^: The classical topic modeling approach that assumes each document contains multiple topics
- Gibbs Sampling Dirichlet Multinomial Mixture^10^: A model specifically designed for short texts, assuming one topic per document

We also include term-weighted variants of these models^8^:

- Log-LDA and Log-GSDMM: Incorporating logarithmic term weighting to reduce the impact of common words
- BDC-LDA and BDC-GSDMM: Using balanced distributional concentration to handle domain-specific stopwords
- Log-BDC-LDA and Log-BDC-GSDMM: Combining both weighting strategies

For transformer-based approaches, we implemented 3 variants of BERTopic with different embedding models from Hugging Face:

- BERTopic-MiniLM: Using all-MiniLM-L6-v2 embeddings
- BERTopic-MPNET: Employing all-mpnet-base-v2 embeddings
- BERTopic-RoBERTa: Based on all-distilroberta-v1 embeddings

These baselines represent a spectrum of approaches to topic modeling, from classical probabilistic methods to state-of-the-art transformer-based techniques, providing a comprehensive evaluation framework for our LLM-based method.

## Results

**Figure 4.**
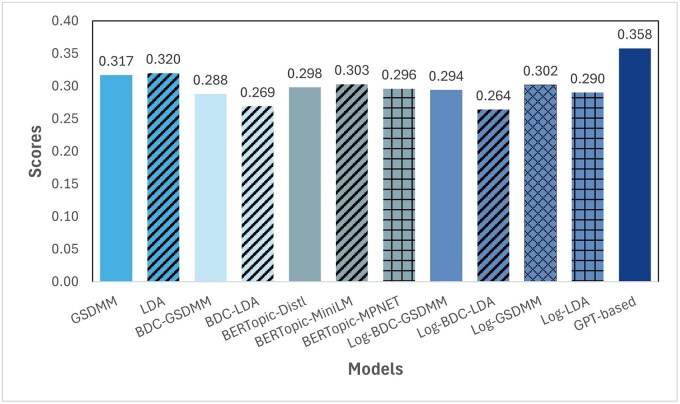
The coherence score comparison between 11 baselines and our method.

Our experimental evaluation demonstrates the superior performance of the LLM-based topic modeling approach compared to traditional methods and their variants. Figure 4 presents the semantic coherence scores across all baseline methods and our proposed approach. The GPT-based method achieves a coherence score of 0.358, substantially outperforming all baseline approaches. This improvement is particularly notable when compared to traditional probabilistic models like LDA (0.320) and GSDMM (0.317), as well as their term-weighted variants, which show coherence scores ranging from 0.264 to 0.302.

The effectiveness of our approach is further validated by the consistent performance across individual batches before the final synthesis, with an average coherence score of 0.354 across the 226 batches. This high batch-level coherence, being close to the final synthesis score of 0.358, indicates that our method maintains semantic consistency not only in the final synthesized topics but also throughout the intermediate processing stages. This consistency suggests that the 2-stage approach effectively preserves semantic relationships while aggregating insights from multiple batches.

The slight improvement from batch-level coherence (0.354) to final synthesis coherence (0.358) can be attributed to the synthesis process’s ability to identify and consolidate semantic patterns across the entire corpus:

1. Local versus global patterns: Individual batches (1000 tweets each) capture local semantic patterns within their limited scope. The synthesis stage examines all 1130 initial topics (5 topics × 226 batches), allowing identification of recurring themes that may appear fragmentarily across different batches.
2. Noise reduction: Words that appear semantically relevant within a single batch but are actually noise become apparent when viewed across all batches. The synthesis process naturally filters these out by selecting only the most consistently meaningful word groupings.
3. Theme consolidation: Related concepts that were separated across different batches due to the arbitrary batch boundaries get reunited during synthesis. For example, emotional terms like “grief” might appear in one batch while “sorrow” appears in another—the synthesis stage can group these together based on their semantic similarity.

The modest improvement (0.004) indicates that our batching strategy successfully preserved most semantic relationships even at the local level, while the synthesis stage provided additional refinement through global pattern recognition.

The transformer-based baselines, including various BERTopic implementations, achieve coherence scores between 0.296 and 0.303, showing that even state-of-the-art embedding-based approaches fall short of our LLM-based method. Notably, the previously proposed term-weighted approaches^8^ show relatively lower performance under our semantic coherence metric, with Log-BDC-GSDMM achieving a score of 0.294, highlighting the limitations of traditional term-weighting strategies in capturing true semantic relationships.

To provide a comprehensive evaluation, we also assessed all methods using the traditional C_v coherence metric, which measures word co-occurrence patterns within the corpus (Supplementary Figure 2, see online supplementary material for a color version of this figure). Interestingly, our GPT-based method achieved a C_v score of 0.500, ranking third overall, while maintaining the highest semantic coherence score. BERTopic variants showed higher C_v scores (BERTopic-MPNET: 0.580, BERTopic-MiniLM: 0.532) but lower semantic coherence. Traditional methods (LDA: 0.421, GSDMM: 0.438) and their weighted variants (0.380-0.465) showed lower performance on both metrics.

This pattern reveals an important distinction: our method prioritizes semantic relationships over statistical co-occurrence. For instance, words like “grief” and “anguish” in our Emotional Distress topic are semantically related but may not frequently co-occur in individual tweets. This semantic focus is particularly valuable for short texts where co-occurrence patterns can be sparse and unreliable. The divergence between metrics confirms that our approach successfully captures conceptual relationships that traditional co-occurrence-based methods might miss.

We instructed our model to generate 5 topics to ensure fair comparison with baseline methods. The extracted topics, their constituent words, and their interpretations by both ChatGPT and human experts are presented in Table 1, with corresponding word clouds visualized in Supplementary Figure 1 (see online supplementary material for a color version of this figure). The topics reveal distinct yet interconnected aspects of the caregiving experience:

**Table 1.** Topics generated by our method.

Topic ID	Top 20 words in topic	ChatGPT label	Human expert label
**1**	Heartbreaking, sadness, grief, tears, sorrow, pain, struggle, difficult, overwhelming, devastating, heartache, anguish, despair, anxiety, frustration, helpless, lonely, burden, stress, worry	Emotional distress	Psychological and emotional burden
**2**	Forget, remember, recognize, recall, memory, confused, lost, disoriented, unfamiliar, forgetful, unaware, misplace, repetitive, vanish, disappear, absent, lapse, slip, fade, oblivious	Memory loss	Cognitive decline manifestations
**3**	Mother, father, grandmother, grandfather, sister, brother, daughter, son, husband, wife, parent, child, sibling, relative, family, bond, connection, support, love, care	Family relationships	Familial roles and bonds
**4**	Feeding, bathing, dressing, cleaning, cooking, shopping, organizing, managing, assisting, supporting, helping, guiding, supervising, monitoring, preparing, arranging, coordinating, handling, overseeing, tending	Daily care tasks	Caregiving activities and management
**5**	Patience, resilience, strength, hope, acceptance, adaptation, flexibility, mindfulness, relaxation, humor, positivity, creativity, distraction, therapy, counseling, support, community, resources, meditation, exercise	Coping strategies	Adaptation and support resources

Topic 1, labeled as “Emotional Distress” by ChatGPT and more comprehensively as “Psychological and Emotional Burden” by human experts, captures the emotional toll of caregiving through terms like “heartbreaking,” “devastating,” and “anguish.” Topic 1 contains emotionally charged terms such as “heartbreaking,” “devastating,” and “anguish,” reflecting the emotional toll of caregiving.

Topic 2 focuses on cognitive decline, labeled as “Memory Loss” and “Cognitive Decline Manifestations” by ChatGPT and human experts, respectively. The word cloud emphasizes terms like “forget,” “disoriented,” and “confused,” illustrating the daily challenges caregivers face in managing cognitive impairment.

Topic 3, identified as “Family Relationships” and “Familial Roles and Bonds,” reveals the complex web of family relationships involved in caregiving. The word cloud prominently features various family roles and emotional connections, emphasizing the collaborative nature of care.

Topic 4 describes the practical aspects of caregiving, labeled as “Daily Care Tasks” and “Caregiving Activities and Management.” The word cloud highlights the diverse range of daily responsibilities through terms like “bathing,” “feeding,” and “coordinating.”

Topic 5, characterized as “Coping Strategies” and “Adaptation and Support Resources,” reflects the resilience and adaptation mechanisms caregivers develop. The word cloud emphasizes both internal resources (“patience,” “resilience”) and external support systems (“community,” “therapy”).

The semantic richness and coherence of these topics, validated by both quantitative metrics and expert interpretation, demonstrate our method’s ability to capture meaningful patterns in caregiver discussions. While we fixed the number of topics at 5 for comparative purposes, future work could explore allowing LLMs to dynamically determine the optimal number of topics based on content analysis.

### Illustrative examples of topic assignment

To demonstrate how our LLM-based approach maps tweet content to extracted topics, we provide representative simulated examples that reflect patterns observed in our dataset:

Example 1—Multiple Topic Assignment: *Simulated Tweet:* “This morning I helped my grandmother with bathing and dressing before cooking her favorite soup. Taking care of her every day is tiring, but the bond we share keeps me going.” *Topic Mapping:* This tweet maps to 2 primary topics—“Family Relationships” (keywords: grandmother, bond) and “Daily Care Tasks” (keywords: bathing, dressing, cooking), illustrating how caregiving experiences often span multiple thematic areas.

Example 2—Cognitive and Emotional Dimensions: *Simulated Tweet:* “Dad looked at me today and didn’t recognize who I was. He asked if I was a nurse. It’s devastating to realize he doesn’t remember me, and the confusion in his eyes broke my heart.” *Topic Mapping:* Keywords like “recognize,” “remember,” and “confusion” map to “Memory Loss,” while “devastating” and “broke my heart” connect to “Emotional Distress,” showing the intertwined nature of cognitive decline and emotional impact.

Example 3—Emotional Burden: *Simulated Tweet:* “It’s so heartbreaking to sit by and watch my mom cry. The sadness and pain in her eyes are overwhelming, and some days it just feels like too much to carry.” *Topic Mapping:* This tweet strongly aligns with “Emotional Distress” through keywords like “heartbreaking,” “sadness,” “pain,” and “overwhelming,” capturing the psychological burden of caregiving.

### Statistical validation

To further validate the robustness of our findings, we conducted 5 independent runs of each method with different random seeds (Supplementary Figure 3, see online supplementary material for a color version of this figure). This additional analysis confirms the statistical significance of our results. Our GPT-based approach achieved a mean coherence score of 0.362 (*SD* = 0.004, 95% confidence interval [CI]: 0.357-0.367), demonstrating highly consistent performance across runs.

Paired *t*-tests revealed that our method significantly outperformed all baselines (all *p*-values < .001). The improvements over traditional methods were substantial: 0.044 points over LDA (95% CI: 0.039-0.049) and 0.050 points over GSDMM (95% CI: 0.045-0.055). Even compared to the best-performing BERTopic variant, our method showed a significant advantage of 0.061 points (95% CI: 0.056-0.066). The narrow error bars and non-overlapping confidence intervals across methods (Supplementary Figure 3, see online supplementary material for a color version of this figure) confirm that the superior performance of our LLM-based approach is both statistically robust and unlikely to occur by chance.

## Discussion and conclusion

In this article, we introduced a novel approach to topic modeling using large language models, specifically designed for analyzing social media content from dementia caregivers. Our 2-stage method, leveraging GPT-4’s semantic understanding capabilities, demonstrates significant advantages over traditional topic modeling approaches and their variants. The superior performance is evidenced by higher semantic coherence scores (0.358 compared to 0.320 for traditional methods) and the extraction of clearly differentiated, meaningful topics that were validated through both automated (ChatGPT) and human expert interpretation.

Our approach successfully addresses several key challenges in social media text analysis. First, we overcome the context length limitations of LLMs through a carefully designed batching and synthesis strategy, processing 224 862 tweets in manageable batches of 1000. Second, we demonstrate that LLMs can maintain semantic consistency both within individual batches (0.354 average coherence) and through the synthesis process (0.358 final coherence). Third, our method produces highly interpretable topics that span 5 distinct aspects of caregiving, each characterized by semantically coherent word groups and validated through multiple interpretive perspectives.

The extracted topics not only achieve higher semantic coherence than existing methods but also provide valuable insights into the challenges and experiences of dementia caregivers. These topics, ranging from psychological and emotional burden to adaptation and support resources, reveal the complex nature of caregiving experiences. In addition to demonstrating methodological advances, our findings translate into several concrete applications. First, health systems could implement real-time dashboards based on our topic-modeling pipeline to monitor caregivers’ public posts and flag emerging crises (eg, surges in “exhaustion” or “overwhelmed” language) for rapid triage by support coordinators. Second, public health agencies and policymakers could track temporal shifts in topic prevalence to identify unmet needs; for example, an uptick in “financial planning” discussions may signal the need for expanded respite care funding or insurance reforms. Third, online support platforms could use caregivers’ topic profiles to match them to appropriate services—directing those discussing cognitive decline to memory-care specialists or those expressing grief to bereavement counseling. Together, these scenarios illustrate clear pathways from our research findings to clinical practice, policy monitoring, and targeted support for caregivers.

From a methodological perspective, this work establishes a new paradigm for using LLMs in topic modeling, demonstrating their potential for understanding complex social phenomena through social media analysis. Our evaluation framework, based on Sentence-BERT embeddings, provides a more meaningful assessment of semantic coherence than traditional co-occurrence-based metrics, offering a new standard for evaluating topic modeling approaches.

Future work could explore several promising directions. First, allowing LLMs to dynamically determine the optimal number of topics based on content analysis could provide more flexible and context-sensitive topic modeling. Second, extending this approach to other healthcare domains and investigating real-time topic modeling of social media content could broaden its applicability. Third, exploring temporal evolution^39-41^ of topics could reveal how caregiving challenges and needs change over time. Additionally, incorporating multi-modal data and developing more sophisticated prompt engineering techniques could further enhance both the semantic coherence and interpretability^42-44^ of extracted topics. Finally, investigating the relationship between different expert interpretations of topics could provide insights into how various stakeholders understand and respond to caregiver needs.

## Supplementary Material

igaf120_Supplementary_Data

## Data Availability

In accordance with ethical guidelines for social media research and user privacy protection, the raw tweet data cannot be publicly shared. However, to support reproducibility and transparency, we make the following resources available: Code repository: The Python code for our 2-stage LLM-based topic modeling approach, including data preprocessing, batch processing, and semantic coherence evaluation using Sentence-BERT, is available at https://github.com/hwq0726/GPT-Based-Topic-Modeling-for-Dementia-Caregiver-Tweets. LLM prompts: The complete prompts used for both stages of ChatGPT topic extraction are provided in Figures 2 and 3 of this manuscript. Topic outputs: The final extracted topics with their constituent words and coherence scores are provided in Table 1 and supplementary materials. Tweet IDs: Researchers interested in the dataset may request access to tweet IDs (not full text) by contacting the corresponding author with appropriate IRB approval and signing a data use agreement. All baseline model implementations and evaluation scripts are included in the repository to facilitate comparison with future work.
