# Supplementary material for: Advanced topic modeling with large language models: analyzing social media content from dementia caregivers
Source: Innov Aging. 2025 Dec 26;9(Suppl 1):S38–47. doi: 10.1093/geroni/igaf120 (PMC12742845; doi:10.1093/geroni/igaf120)
Supplement: igaf120_Supplementary_Data [file igaf120_supplementary_data.zip › innage suppl He et al.docx]

# ***Innovation in Aging* Supplementary Material: He et al.** **Advanced Topic Modeling with Large Language Models: Analyzing Social Media Content from Dementia Caregivers.**


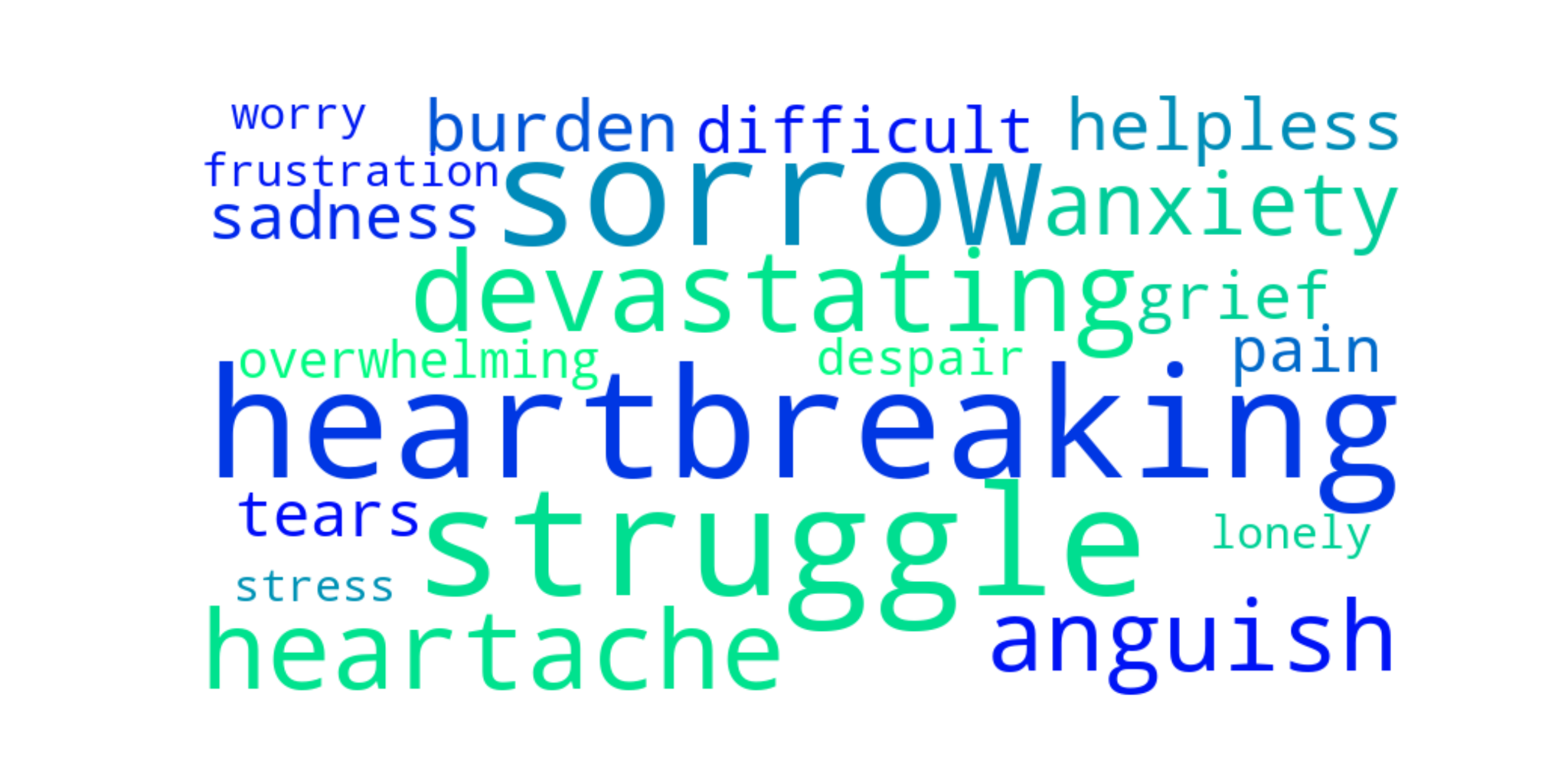

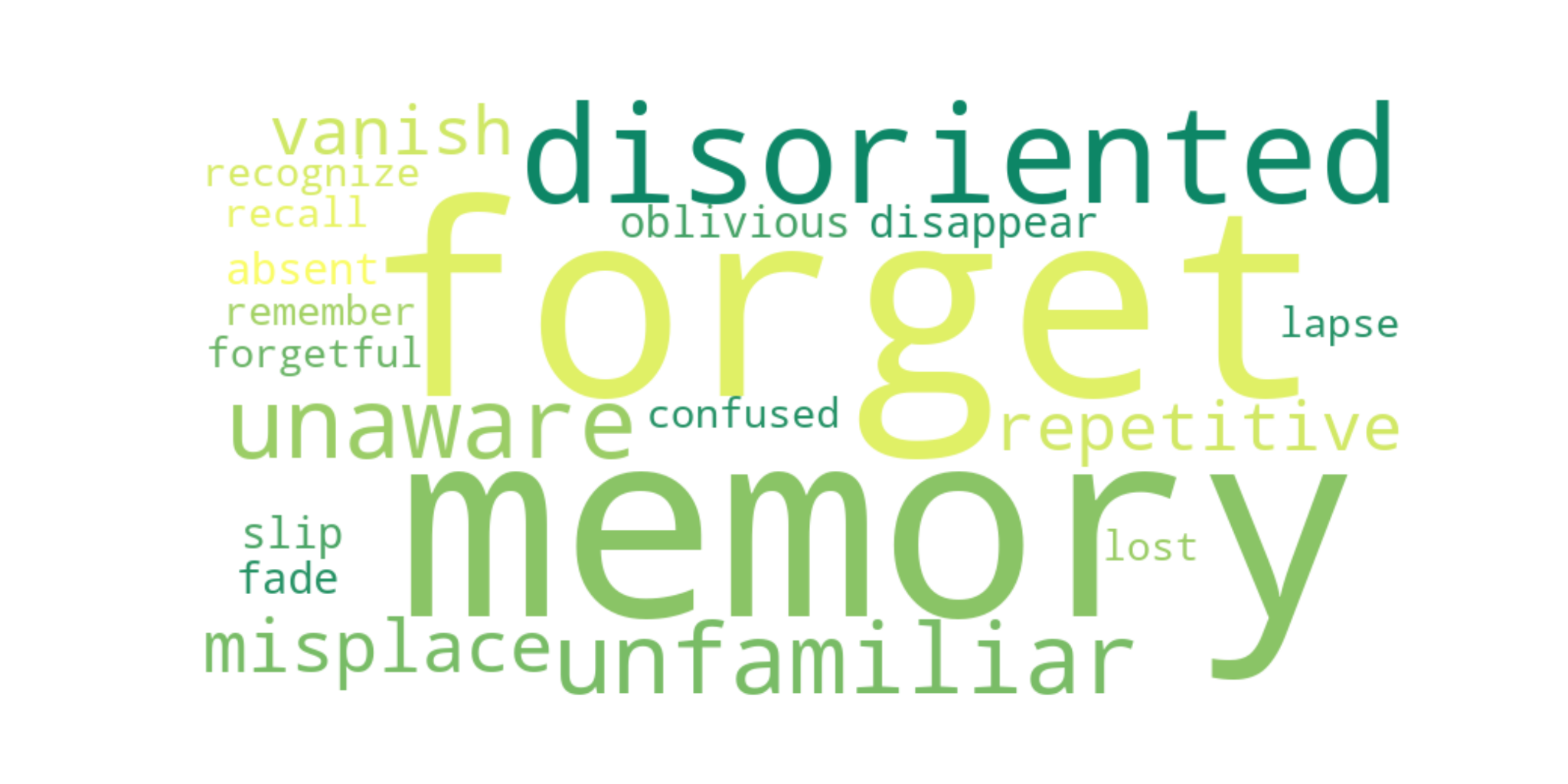

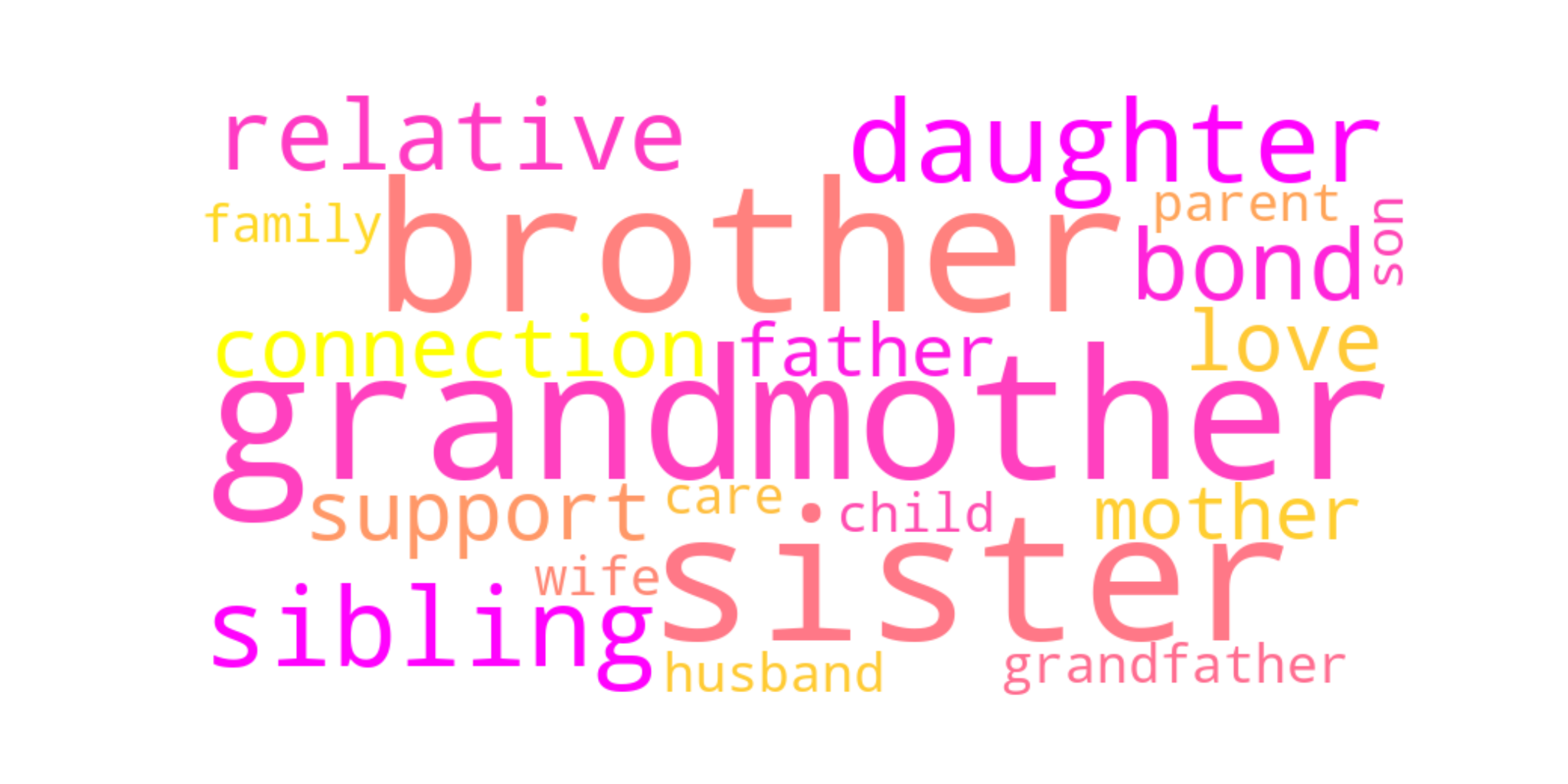

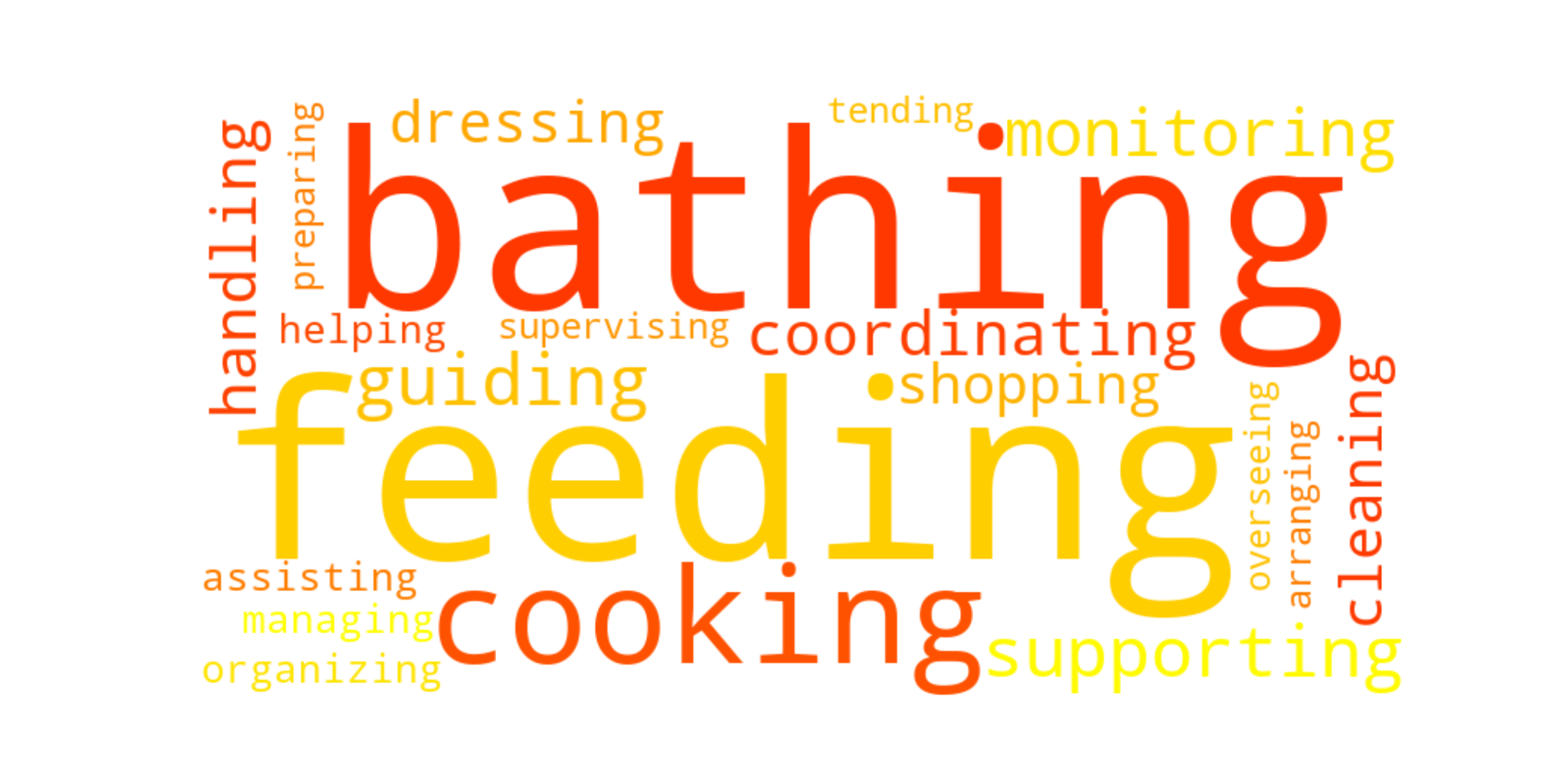

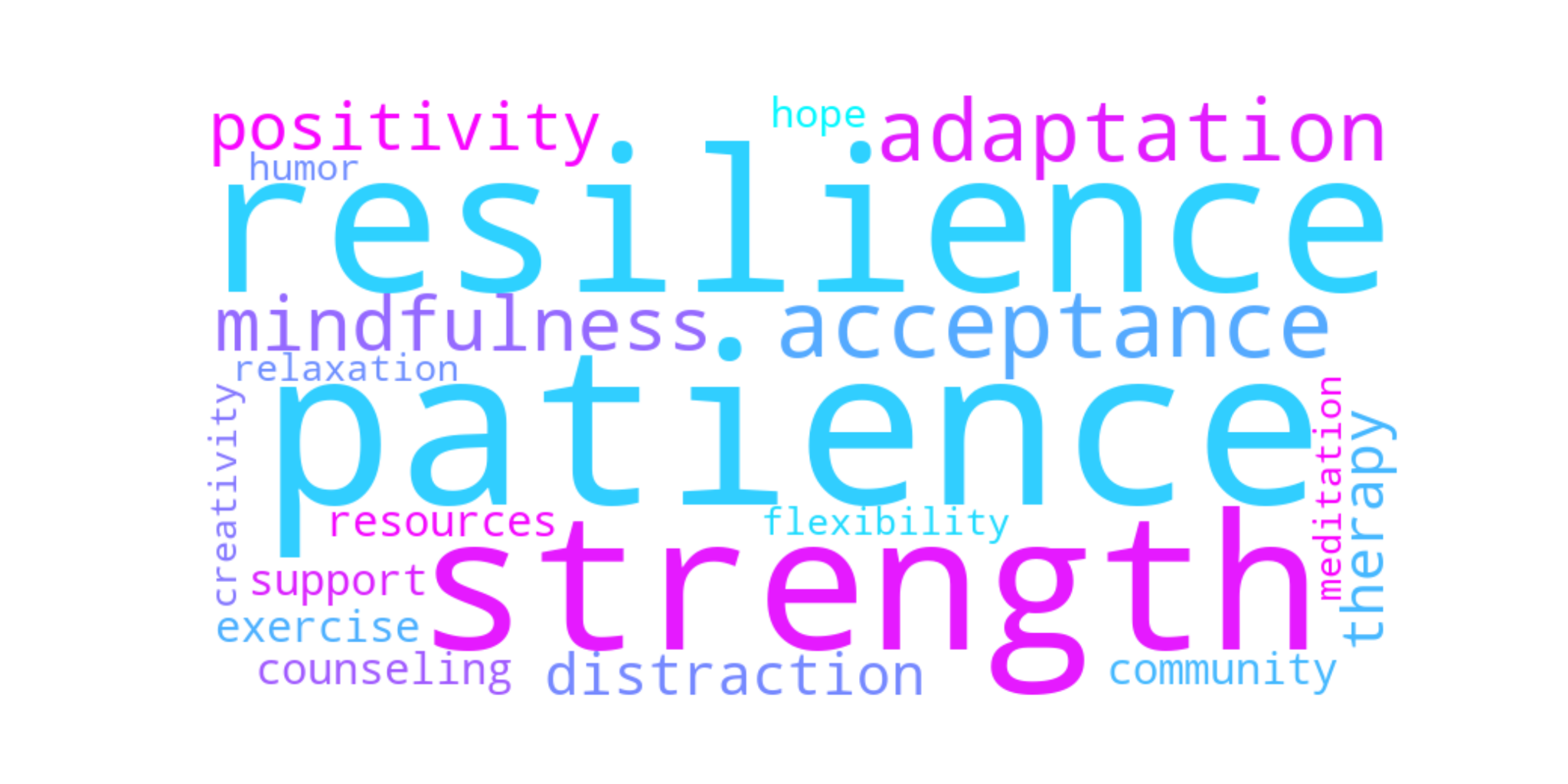


**Supplementary Figure 1:** The word clouds with respect to five topics produced by our method.


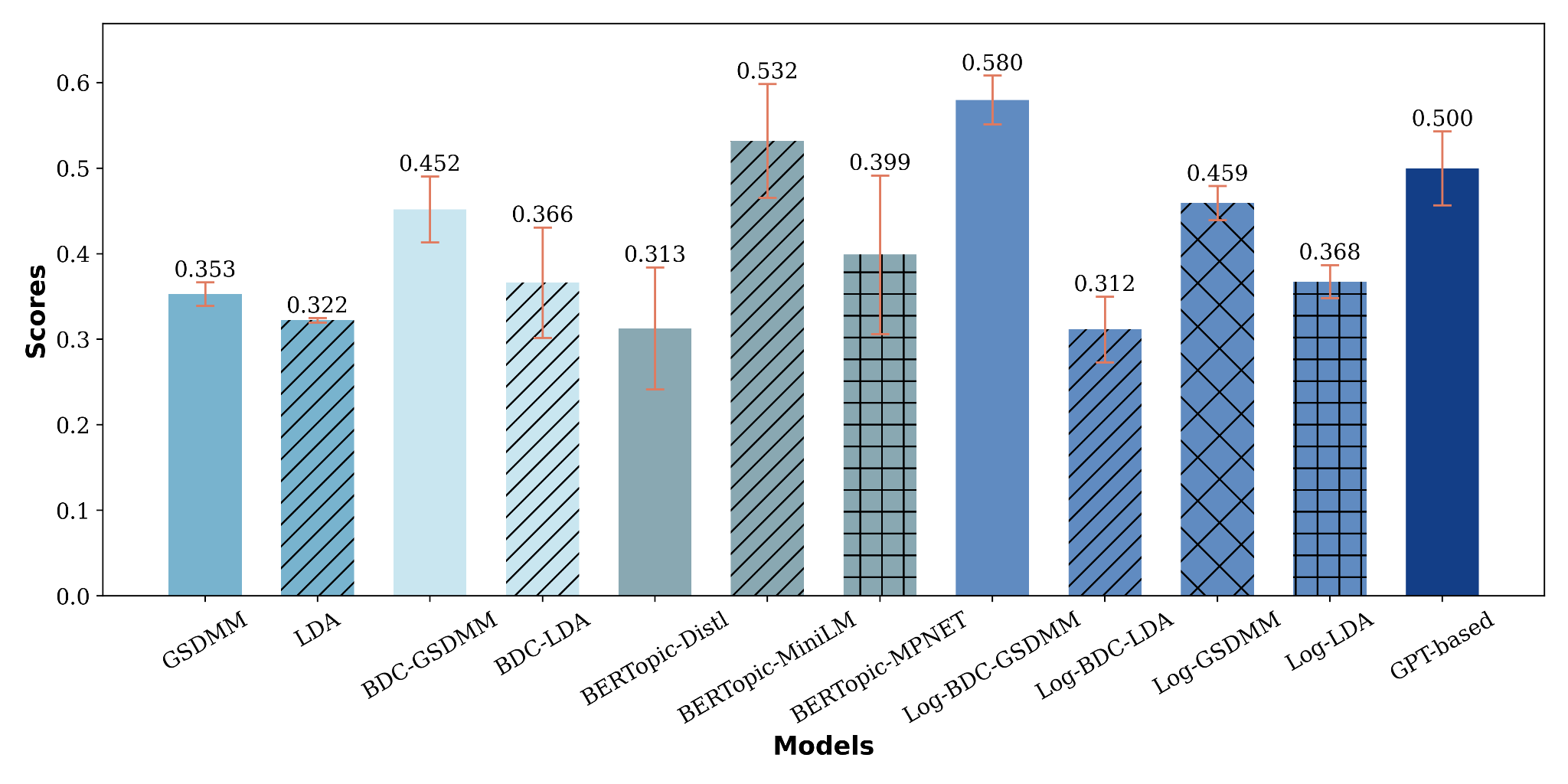


**Supplementary Figure 2:** Comparison of traditional C_v coherence scores across all methods. While BERTopic variants achieve higher C_v scores, our GPT-based method maintains competitive performance (0.500) while excelling at semantic coherence (see Figure 2).


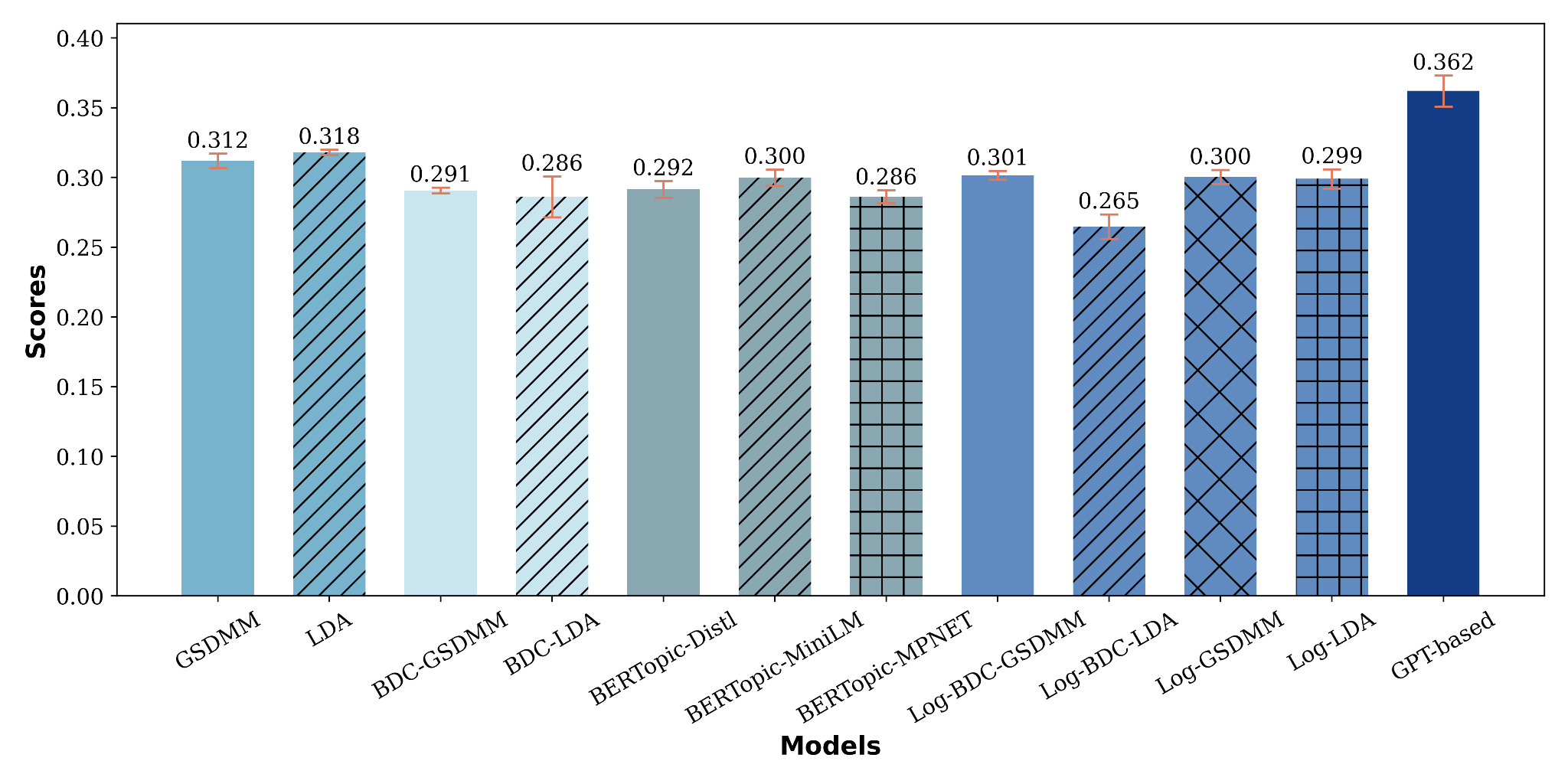


**Supplementary Figure 3:** Semantic coherence scores across all methods based on 5 independent runs with different random seeds. Error bars represent standard deviations. Our GPT-based method (0.362 ± 0.004) significantly outperformed all baselines (all pairwise t-tests p<0.001), demonstrating both superior performance and robustness.
